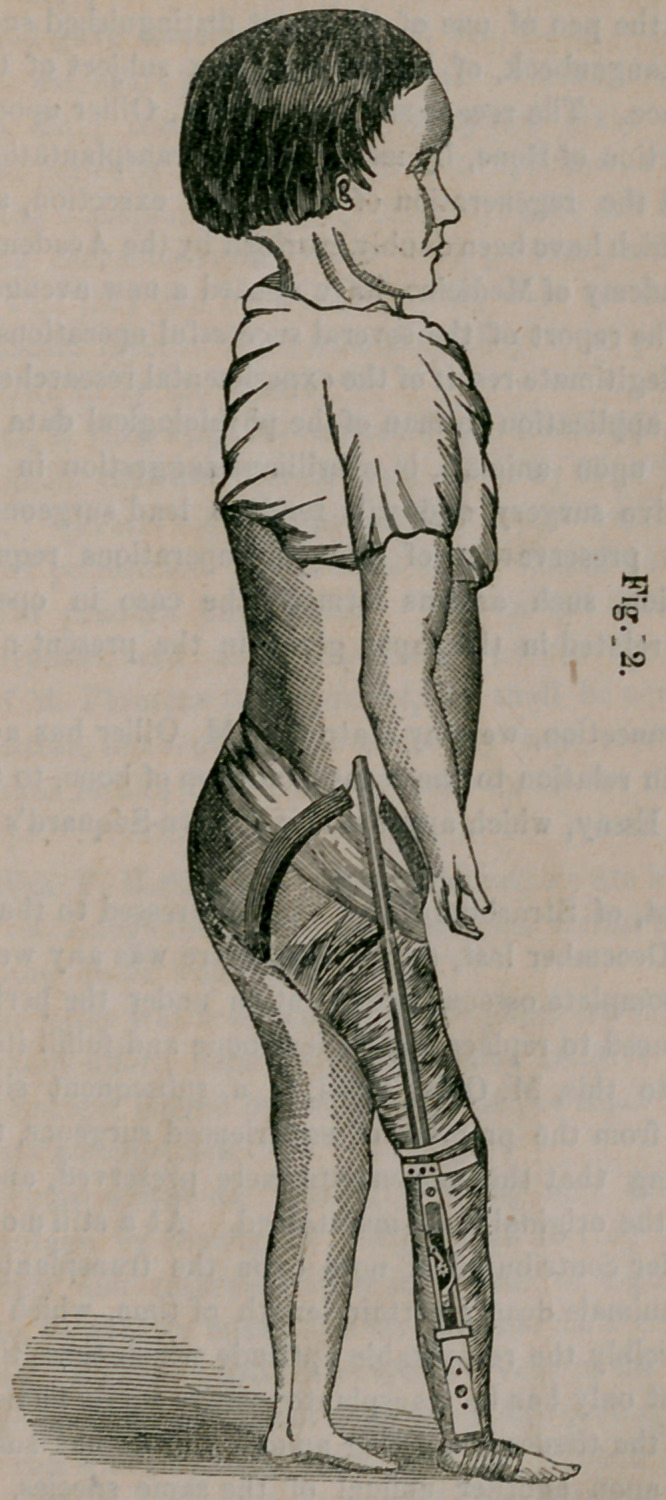# N. Y. Medico Chirurgical College—Discussion on Morbus Coxarius

**Published:** 1860-04

**Authors:** James Bryan

**Affiliations:** Chairman


### PROCEEDINGS OF SOCIETIES.

*Medico-Chirurgical College.* January 26, 1860. Dr. James Bryan, Chairman.

Dr. Lewis A. Sayre, who was appointed to read a paper on *Morbus Coxarius*, stated that he had not prepared a paper on the subject, but would make a few remarks upon the disease, and explain an apparatus which he had contrived, to be used in the treatment of the same during its earlier stages. He divided the disease into three stages, as follows:

The *first* is the inflammatory condition of the disease, having its origin from a fall, blow, injury, or other extraneous accident, which will induce a synovial inflammation of the hip-joint, and occurring in a strumous constitution, will go on, unless arrested, until the cartilage and bone are involved; finally terminating in what is called morbus coxarius, and resulting in the deformity, anchylosis, or shortening which are peculiar to this disease—or death. The symptoms in this stage, such as pain in the knee, imperfect flexion, pain or pressure of the trochanter, or percussion of the knee, &c., &c., he would not dwell upon, as they were familiar to all. He would merely remark upon one symptom—that of pain produced by pressure, bringing synovial membrane in contact—that it was a remarkable fact, that in the healthy condition the synovial membrane bore pressure, without the least sensation, as proved by jumping from great heights and striking upon the heels, producing no pain whatever. Yet when this membrane is inflamed, the slightest amount of pressure produced the most intense suffering; and hence the necessity of *removing all pressure* from this delicate and sensitive membrane, when in a state of inflammation. This inflammation, unless arrested, will produce effusion, and if it be of much amount, will produce a *peculiar* deformity, viz.: an *apparent* elongation of the limbs; *eversion* and *abduction;* flattening of the nates; the rima nates on the diseased side being lower than on the sound side; flexion of the thigh on the pelvis, and the leg slightly flexed upon the thigh. If the effusion be excessive, or the inflammation acute, you will have an *apparent* anchylosis, caused by muscular contraction, which is an involuntary act, produced by reflex action of the inflamed or irritated nerves, *and is done for the purpose of keeping the joint perfectly still*; which is the indication for the surgeon to imitate this action, and accomplish this result, of *perfect rest* to the inflamed synovial membrane, by artificial means, and thus save the necessity of this *constant* muscular effort, which, continuing day and night for months, eventually exhausts the system and brings on hectic fever.

The flexor muscles of the thigh, the tensor vagina femoris, the pectineus and rectus femoris are so firmly contracted that the whole pelvis moves on the opposite acetabulum; and you will distinctly see the ilium of the opposite side move, upon any attempt to rotate, adduct, or abduct the diseased limb. Even under chloroform this motion takes place, as I have seen in several instances.

This does not depend upon true or bony anchylosis; for by division of the flexor and adductor muscles, or by puncture of the joint, you will have free motion of the limb, showing that there has been no bony anchylosis. This is the *second* stage of the disease, and if it be not arrested, ulceration of the capsule takes place, and the fluid, whether pus or plastic lymph, becomes effused in the surrounding tissues, and in the majority of instances burrows in various directions, and finally produces an external opening—in different portions of the thigh—sometimes only one, but frequently more, and in many instances some distance from the affected joint. The capsule being thus ruptured, we have the *third* stage of the disease, and the peculiar change that takes place in the deformity occurring so suddenly has led to the false idea that a luxation had taken place. The limb is now apparently shorter, adducted, inverted, flexed in *hip only*—pelvis raised—projected backward; in fact, the position is almost the reverse of what it was in the second stage, and thus occurs *suddenly* upon the perforation of the capsule.

| Second Stage. | Third Stage. |
|---|---|
| Limb (apparently) longer. | Limb (apparently) shorter. |
| " abducted. | " adducted. |
| " everted. | " inverted. |
| " flexed in both joints. | " flexed in *hip* joint only. |
| Foot touches the ground with sole. | Foot touches with ball only. |
| Pelvis lowered on diseased side. | Pelvis raised on diseased side. |
| " projected forward. | " projected backward. |
| Nates low and flat. | Nates high and round. |
| Pain most intense. | Pain greatly diminished. |

TREATMENT.

In the treatment of this disease, in its first stage, local depletion, by leeches or cups, is often necessary, with a relaxed condition of the bowels. But the most important of all, and on which all prospect of success will depend, is *rest of the joint*, and *perfect freedom from pres-*

*sure of the synovial membrane*, together with such constitutional remedies and general support of the system which we find requisite in all strumous diseases. The use of issues, at this stage of the disease, as formerly employed, he condemned, as they often aggravated the disease, if they did not hasten the death of the patient. When left to itself, the rest which is so essential to the joint is procured by the firm muscular contraction which prevents the motion in the joint, and is so perfect, as in many instances to assume the appearance of genuine bony anchylosis. But this *constant muscular contraction* exhausts the nervous system, and induces hectic fever; gives the child nocturnal spasms, of intense agony; prevents nutrition of the limb, which results in atrophy. We therefore resort to artificial means to produce this rest, and divide the firmly contracted muscles, to prevent the head of the bone from being pressed against the acetabulum.

I have treated a number of cases in this way, after the plan of Dr. Bauer, of Brooklyn, with the happiest results; using as a means of rest and extension his " Wire Breeches," an apparatus for the treatment of this disease, in some of its stages, which is far superior to any other that I have ever seen or heard of.

The great difficulty to be overcome in securing *entire rest* of the joint, is the danger of producing bony anchylosis, resulting from the general inflammation of the surrounding structures, and sometimes great stiffness and partial anchylosis of the well joints. On the other hand, if we remove the instrument, and commence passive motion, *without keeping up extension*, there is danger of reproducing the inflammation.

I have, therefore, come to the conclusion that *perfect rest*, however essential it may be to an *inflamed synovial membrane*, is not only unnecessary to the ligaments, (which, in the earlier stages, were not involved in the inflammation,) but was positively *physiologically wrong*. As the eye needs the healthy stimulus of light, so does the ligament need the stimulus of *motion*. If, then, we can give motion to the ligaments of a joint, while at the same time we prevent muscular contraction, so as to remove pressure from the synovial membrane, we shall accomplish our object, and at the same time give the patient the benefit of out-door exercise, which is so essential in this disease.

He has tried to make an instrument by which the muscles can be overcome, while at the same time a certain amount of motion is given to the limb, and practically it answers every purpose. In the few cases in which he had used it, it has answered more than his expectations, the patients begging to have it reapplied whenever it has had to be removed, for repairs or any other purpose.

The instrument shown was constructed under his direction, by Mr. FORD, of No. 85 Fulton Street, a most ingenious mechanic, and one of the few surgical instrument makers who is willing and able to practically construct what you may originate or devise. The instrument consists of a steel bar, one and a half inches wide, (see cut,) extending from above the ilium to the external malleolus, slightly curved inward at its upper extremity—at its upper extremity there is a ball and socket joint, (concealed at *e*, visible at *f*,) resembling the hip-joint, and a pulley over which plays a catgut, which is fastened at either end to a piece of firm and strong india-rubber tubing, (*d*,) which passes around the groin, and makes the means of counter-extension; adhesive plaster spread upon firm inelastic cloth, about two or three inches wide, extends from the trochanter major to the external malleolus, and is closely secured to the limb, by plaster and a roller, for the purpose of extension. This plaster is sewed fast at its lower extremity to a piece of webbing or belt, which plays over a roller in the lower extremity of the steel shaft, (*a*,) and is firmly secured by a buckle a few inches above its lower extremity. The steel shaft consists of two pieces, the one sliding into the other, and capable of being extended at pleasure, by a ratchet and cog-wheel, (*b*,) worked by a key, and retained at any position required, so that the extension and counter-extension are perfect, and entirely within the control of the surgeon. There is also a knee-cap (*c*) and strap intended to pass over the knee, and around the limb, to assist in sustaining the limb in position.

As the disease is one which requires many months for its treatment, it is necessary to keep up the extension, and increase the length of the instrument just in proportion to the growth of the child. As children generally grow from three to four inches in a year, or at the rate of about 1-16th of an inch a week, the cogs and ratchets are cut so that they extend the instrument that distance at every single turn. The surgeon can therefore move it one cog every week, more or less, as his judgment may dictate. By the use of this instrument with leeches and iodine to the joint, combined with constitutional treatment and support, I have seen cases terminate before passing to the second stage, and recover without deformity, and with perfect motion.

When the disease has gone on to the *second stage*, and there is extensive effusion in the joint, which cannot be absorbed by the use of iodine or blisters, I should then puncture the joint, and allow the parts to resume their natural position, and then apply the instrument; by which means the necessity of dividing the tendons will be avoided in many instances, as the india rubber, by the *permanency* of its contraction, will overcome the muscular rigidity. At least it has done so in the few cases in which he has tried it.

If it did not, he would at once resort to myotomy, as nothing will so quickly relieve the agony of the patient as the division of the contracted muscles. Another advantage derived from puncturing the joint is in our diagnosis: when the joint is distended, we can get no crepitus, and cannot tell whether the cartilages are destroyed, and the bone is bare or not; but as soon as punctured we can ascertain the fact with positive certainty. And if the disease has so far progressed that you get *positive bony crepitus*, in other words, you have dead bone within the ligament, he is positively convinced from experience that the only proper treatment is immediate *exsection* of the dead bone. All delay, after you get *bony crepitus*, is but to the detriment of the patient; for if you wait too long there is danger that the acetabulum will be perforated, and pus escape into the pelvis. The sooner exsection is performed the better, and the advantage we gain in diagnosis, by puncturing the joint, more than compensates for the risk of the operation. In fact, done with proper care, there is not much risk. Dr. Bauer, of Brooklyn, has punctured the hip-joint now more than fifty times, and as yet without a single accident.

The operation of exsection is attended with no especial danger, and almost no hæmorrhage; it is a trifling operation compared with exsection of the knee or elbow. The results of the operation have been very remarkable indeed, when we consider the circumstances under which they have been performed. All the fatal cases that he had been able to trace had been performed too late; the acetabulum perforated, fragments of bone left behind; and in one instance no extension was used, and the sharp end of the femur perforated a blood-vessel, causing death by secondary hæmorrhage. In cases where the operation has been performed at the proper time, and the proper treatment pursued afterwards, the success has been very remarkable; in many cases the patient recovering with considerable motion of the limb. The patient on whom he performed this operation, the first ever performed in this country, now some seven years since, he saw not long since *jumping a rope* as lively and strong as any child. She has only about an inch or an inch and a quarter of shortening, and has every motion of the joint perfect and complete.

Dr. O'Reilly remarked that he agreed entirely with Dr. Sayre in condemning the use of issues in this disease, and had endeavored to point out the mischief resulting from their application, in a paper published in the *Medical Gazette.* He would also state, that he was in the habit of treating this disease successfully, in many cases, without deformity. He depended mainly upon the plan of treatment laid down by Dr. O'Burns, of Dublin, in 1843—namely, the use of mercury. Dr. O'Burns also recommends that the patient should be kept in a quiet position from four to six months, but does not advise anything in the shape of a splint. When the disease has advanced to the second stage, Dr. O'Reilly combines with the mercurial some *tonic* remedy, such as the compound tincture of bark, together with cod-liver oil, so as to remove the scrofulous taint, and build up the system. Under this treatment, he thought that most of the cases improved, while some recovered with almost no appreciable lameness. In one case, that of a little girl, put under this treatment, the recovery was complete; she is now a fine, stout, healthy girl, and has scarcely any deformity whatever.

Dr. Raphael thought that too little importance had been attached to the use of the bichloride of mercury in this disease. He considered it as one of the most valuable remedies in the whole Materia Medica, not only in this disease, but in many others in which it was formerly used, where it now seems to have been abandoned. Secondary syphilis is one of these; he considered it as a most valuable remedy in this affection. With regard to morbus coxarius, he would say that he himself had had the disease, and was treated with the bichloride of mercury, which perhaps might account for his strong partiality for this remedy. He knew very little about the history of his case, except what he had learned from his parents; and their statements led him to believe that the disease advanced to the second stage, as he was confined for some time. With regard to the present condition of the joint, he thought that he had a subluxation, resulting from partial absorption of the acetabulum, so that the head of the bone rests upon its upper edge. When he moved the affected limb, he could feel the head of the bone slip out of the cavity. He is unable to cross the leg completely, but has anterior and posterior motion perfectly. There is about one-half inch shortening, which he has remedied by placing an inside sole in the boot of that side. To the local treatment in his own case he did not attach much importance; he considered that the beneficial results were almost solely attributable to the constitutional treatment employed; he could not, therefore, viewing not only his own case, but many others, avoid being impressed with the necessity of using constitutional remedies, and more particularly the bichloride of mercury. Dr. Raphael then requested Drs. Sayre and Carnochan to examine his case, to see if the diagnosis was correct.

Dr. Gardner then remarked that he was prepared to place great faith in the apparatus of Dr. Sayre, from what he had seen of treatment of diseases of the spine by the instrument of Dr. Davis, which is so constructed that, while it permits a certain amount of motion, it prevents the two bodies of the vertebræ from pressing against one another; thus permitting the patient to take exercise. He had also used the bichloride of mercury in this disease, and had more faith in it than most other preparations.

Dr. Sayre, who meanwhile had examined the case of Dr. Raphael, stated that in his opinion there was no subluxation, or destruction of the joint. The half inch of shortening he thought resulted from a lack of nutrition in the affected limb, during the active stage of the disease, while the other went on increasing in size; a circumstance which he had noticed in other cases. There is some grating motion in Dr. R's case, and it is quite probable that the cavity of the acetabulum is partially filled with plastic effusion, preventing the head of the bone from fitting the cavity, and as it rises over these points, gives him the sensation of subluxation.

Dr. Selden stated that he had used the bichloride of mercury in the hospitals on Blackwell's and Ward's Islands, and had seen no great benefit result from its use. He gave the preference to iodide of potassium, even where the patient has had syphilis in early life.

Dr. O'Reilly did not consider this any argument against its use in morbus coxarius.

Dr. Carnochan remarked that, in a disease like morbus coxarius, which naturally runs through a period of time ranging from six months to two or three years, you can scarcely expect to bring the treatment of the disease to a unit. Morbus coxarius is in fact a series of symptoms, and to say that issues or any other form of treatment is not applicable to the disease, *may* or *may not* be true. It may depend upon the stage of the disease in which this particular treatment may have been tried, whether it failed or succeeded. Morbus coxarius begins as an acute disease, as a phlegmasia, and is to be treated as such. It is, however, associated with a taint which makes a difference between it and ordinary inflammation. There must be, then, a treatment for this peculiar phase of the disease. The disease at this stage, if left to itself, may end in resolution, or it may go on to the formation of pus. Here, then, we cannot say that *one particular* form of treatment is *always* applicable; and so it is with the third stage. So, when we speak of the treatment, we must also speak of the *particular condition* to which this treatment is applied. Hence I think that a great many mistakes have been made with regard to the treatment of morbus coxarius, and hence the confusion with regard to the essays which have been written upon it. The two great principles at issue with regard to treatment of morbus coxarius are: one, to secure rest by mechanical means; the other, to allow the patient to follow the laws of instinct. I think in such cases the more favorable plan is to allow the patient to follow his own instinct, without the use of any apparatus whatever. In some twenty cases where the patients were allowed to follow their own instincts so far as motion was concerned, the result was favorable; they all got well. The plan of Dr. March, of Albany, is to draw the head of the bone from the acetabulum, and then confine the patient to bed. I know of two cases treated in this way, and in both the treatment failed to bring the disease to a successful issue. The discussion of this subject, so far, has been too *general.* One gentleman tells us that the application of issues is wrong, while Dr. Sayre says that the muscles must be divided. What we want is the *phase* of the disease to which these different kinds of treatment are applicable. So far as the nature of this affection is concerned, I have been in the habit of looking upon it as a constitutional disease. It is a strumous affection; I would almost regard it in the same light as strumous ophthalmia. I have consequently, in the treatment, relied very much upon constitutional measures. In the first stage of the disease, as much quietness of the parts as possible should be enforced; as a general rule, the patient will, if left to himself, assume instinctively that position which will give rest to the joint. Dr. Physick was in the habit of applying an apparatus for the purpose of keeping the joint at rest. At the present day, we have the apparatus of Dr. March, which is recommended by some; then we have Dr. Sayre recommending puncture of the joint. These methods are but matters of experiment as yet. So far as the discussion has gone, we have no precise data. I might mention, as still leading to further discussion, the usual plan of treatment which I have adopted. In the first stage of the disease, I now use mechanical means of a gentle character, such as splints of *pasteboard*, for the purpose of maintaining the joint at rest. The joint is kept at rest in this manner for three or four months; in the mean while I deplete, or rather *revulse*, by acting on the intestines with doses of rhubarb, soda, and ipecac; at the same time I give the child such a diet as can be taken and *digested*, but do not attempt to *force* upon it any form of nutritive material. I order such articles in the way of food as eggs, beef-tea, soup, etc., in just such quantities as the *child will partake of them;* and if this stage ends in resolution, there is an end to the case. I have been fortunate enough to have this occurrence take place in many cases..

Sometimes, however, no treatment appears to be of any avail; the case then progresses to the suppurative stage. *Now* is the time for counter-irritation; the disease is now chronic. The irritable pulse of the inflammatory stage is not present; you want then some counter-irritation applied near the part, not sufficient to increase the *general* irritation; not to make a point of irritation from which there will be communicated a general irritation to the whole system, but just enough to act as a *flux* from the diseased part. There is no doubt that the old doctrine, "*ubi irritatio ibi fluxus*," is true I think that all experience will prove that *that* is correct. I usually effect the counter-irritation by means of the "actual cautery," applying it midway between the tuberosity of the ischium and the trochanter major. In the first stage we deplete, to revulse upon the joint; but in the second stage, where the disease has become passive, where suppuration is about to take place, or where it *has* taken place, counter-irritation is to be resorted to; it must not, however, be carried too far; you cannot afford to draw away so much as to prostrate the general vitality, but just enough to produce a flux from the part. The second stage may be treated successfully, or it may not. If *not* treated successfully, what takes place? You have pus forming in the joint; you have the capsular ligament perforated by ulcerative absorption, and matter finding its way into the joint; you have abscesses forming round about the joint. Here is another phase of the disease; what are you to do now? Are you to continue the issues, or are you to leave them off? Are you to open the joint or not? These are knotty points in private practice; for when the joint is opened and the matter let out, even in the most cautious manner, we often have irritative, and finally hectic fever, setting in; and we have such men as Abernethy laying down rules by which this irritative fever may be prevented, thus recognizing it as a consequence of the operation. Again the question comes up, Shall we open the joint freely, or give exit to the matter by subcutaneous puncture? These are points perhaps not yet *permanently* decided. Will a section of the muscles have much effect in this second stage? How many muscles do you have to divide? Not only the rectus, gracilis, sartorius, and other muscles in front, but also those which run from the tuberosity of the ischium to form the ham-string and the gluteal muscles. It may be very well to ask what is to be the influence of this traumatic lesion from cutting so many muscles? But let us go on to the third stage, where the disease is still, and attended with no effusion at all, and with very little pain, the patient being perhaps able to jump about, or even to run about the streets. Here we have the matter running from sinuses formed about the hip-joint. Here is another phase of the disease, and no one supposes that issues will be of any benefit at this period of the disease; so when we speak of morbus coxarius, we must take it up seriatim, not as a unit. Exsection has been spoken of. I can imagine very well that in certain periods of the third stage the head of the femur may be cut off with advantage; but are we always to be in a hurry to cut off the head of the femur? I should say it would be better to wait a little before we undertake such a serious operation, because the disease sometimes goes on much more favorably than we supposed it would, if we let it alone. The doctor has also spoken of puncture of the joint in the early stage, and subsequent application of the instrument. So far as puncture of the joint is concerned, I should hesitate very much to perform it in private practice, for fear that inflammation of the joint would follow.

Dr. Sayre said it was chiefly in the inflammatory stage that he expected benefit from this instrument, applied so as to get freedom from pressure on the infra-synovial membrane, while at the same time you give the child the benefit of exercise. Some of the treatment as laid down by Dr. Carnochan, varying according to the several stages, is very properly and very judiciously viewed, particularly as he now recommends the pasteboard splint in the early stages for some months, to keep the limb quiet, rather than to leave the patient to the *instincts* of nature; but he (Dr. S.) was not prepared to entirely agree with him in some points. He asks, if the disease goes on to the second stage when there is effusion in the joint, whether it would be well to puncture. He should reply, if we fail entirely in producing absorption of the fluid by the use of remedies, then, rather than have the joint perforated by ulceration, he would make a valvular puncture, and withdraw the fluid, and would have little fear of constitutional symptoms. Of course, the operation is attended with some danger, as are all operations, no matter how slight, in children. In regard to division of the muscles, the necessity of dividing the gluteal has been referred to. This, he said, was not exactly according to his experience. In the second stage, the thigh is flexed upon the pelvis by the rectus femoris, and abducted, lifted outward by the tensor vaginæ femoris. The gluteal muscles exercise almost no influence. On examining the gluteal region, you find the nates flattened, and the muscles loose and flabby. In some cases we find the tensor vaginæ femoris so firm and hard, that it almost obliterates the ant. sup. spinous process of the ilium, the difference on the two sides being very marked The pectineus and gracilis will be found very firmly drawn in the other direction. To restore the limb to its position, it is only necessary to divide *those muscles* which are *firmly* and *rigidly* contracted. The treatment by division of the muscles, in cases where the patient is suffering the most intense pain, where the disease has gone on to the suppurative stage, has been followed by the most perfect relief from all *pain*; the suppuration, of course, goes on as before, but *sleep* can be had without anodynes; the appetite is restored, and the general condition of the patient improved.

In the third stage of the disease, no one would attempt to get into the joint by puncture. Where the joint is destroyed, where naked bones come in contact, and pus is endeavoring to escape, rather than let it alone, he would make an incision *fairly* and *fully into* the joint; no valvular incision, but one by which the air can get in and *out* again; nothing need be feared from the introduction of air, for it is a *joint* no longer —it is no more entitled to the name of a joint than any other portion of dead bone, and he would therefore cut down to it, and remove it.

Dr. Carnochan remarked that he did not consider the bone as *dead*, but only in a *carious* condition. If the bone were dead, it would assume the character of a sequestrum, and be thrown off. Unfortunately the bone is still living, but carious. We know very well that caries of bone ends in resolution; we see this in the spine, where, in the course of time, the disease terminates by bony anchylosis. We have this same condition of things about the joint; the bone is in a carious condition. The question is, whether it is proper to cut away the head of the bone, or to leave the case to the recuperative power of the system, influenced by therapeutic means, properly directed? Again, if the head of the femur were situated like the head of the humerus, the operation might be more practicable, but we cannot chisel out the bone as we wish without being liable to create suppurative inflammation of the pelvic structure. He would therefore be unwilling to adopt this practice, because we know the disease will get well with anchylosis; sometimes the surfaces become ebernated, forming a sort of a joint, and allowing motion even after the third stage.

Dr. Sayre.—If this dead bone is allowed to remain, there is danger that it will create disease in the acetabulum, and then the case is almost hopeless, and the operation would be, perhaps, followed by fatal results. The operation, to be of benefit, *must be performed* at the *proper time*. One more point—he thought the removal of the dead bone from the hip-joint is just as proper as the removal of the head of the humerus, and certainly more necessary. The acetabulum is a delicate structure, thin in its inner and pelvic wall, and may become perforated —a piece of dead bone may remaim in the glenoid cavity for years without producing any serious result; not so, however, with the acetabulum.

Dr. Carnochan asked Dr. Sayre what he would do in the case of a child of about 12 years of age, who has passed through the different stages of the disease, and is now in the following condition: The tissues about the part are somewhat thickened; there are two sinuses leading to the diseased joint, which throw out, not exactly pus, but sero-purulent matter in no very great quantity. The child is able to be up, walks about, sleeps well, the limb is slightly bent inward, the head of the bone seems to be relieved in a certain position, and the child instinctively assumes it. There is no anchylosis. This is a case which might get well without treatment other than therapeutical.

Dr. Sayre.—Did you find crepitus?

Dr. Carnochan.—Crepitus could not be detected, but the probe leads down to dead bone.

Dr. Sayre.—If dead bone could be found, and I could not remove it by dilating the sinuse with compressed sponge, I would cut down and remove it, just as I would anywhere else. In many of these cases, there is an inflammation of the periosteum of the femur, and no implication of the joint; the periostitis and necrosis, involving only the upper portion of the femur, the joint, proper, having entirely recovered its healthy action; in these cases, I presume Dr. C. would make no objection to exsection of the dead portions. He could see no reason why the same rule should not apply, where the *dead portion is embraced within the capsular ligament*; but, on the contrary, every reason why it should be *more speedily* removed, than where it only involved the shaft of the bone; for, within the capsule, it endangered the perforation of the acetabulum, and consequently the pelvic viscera.

Fig. 1.

Photographs taken by Mr. Gurney, of a case of morbus coxarius, with and without the instrument applied, were then shown to the College. The accompanying cuts were reduced from these photographs. Fig. 1 shows the malformation, and the position the patient naturally assumes in the first and second stages of the disease. Fig. 2 is the

Fig. 2.

same case with the instrument applied; shows the method of its application and the improved position of the patient, as the result of the use of the instrument. The two photographs were taken within twenty minutes of each other.

## Figures and Tables

**Figure f1:**
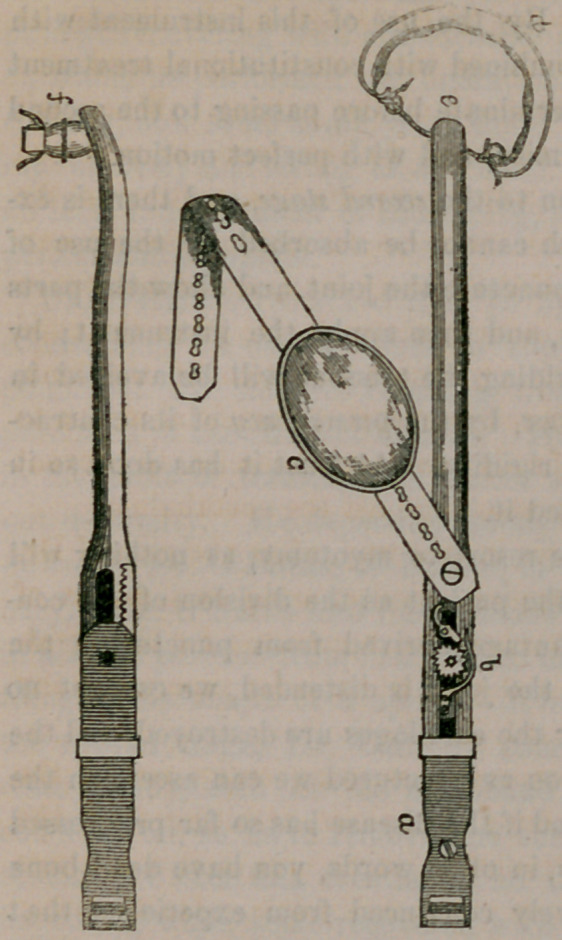


**Fig. 1. f2:**
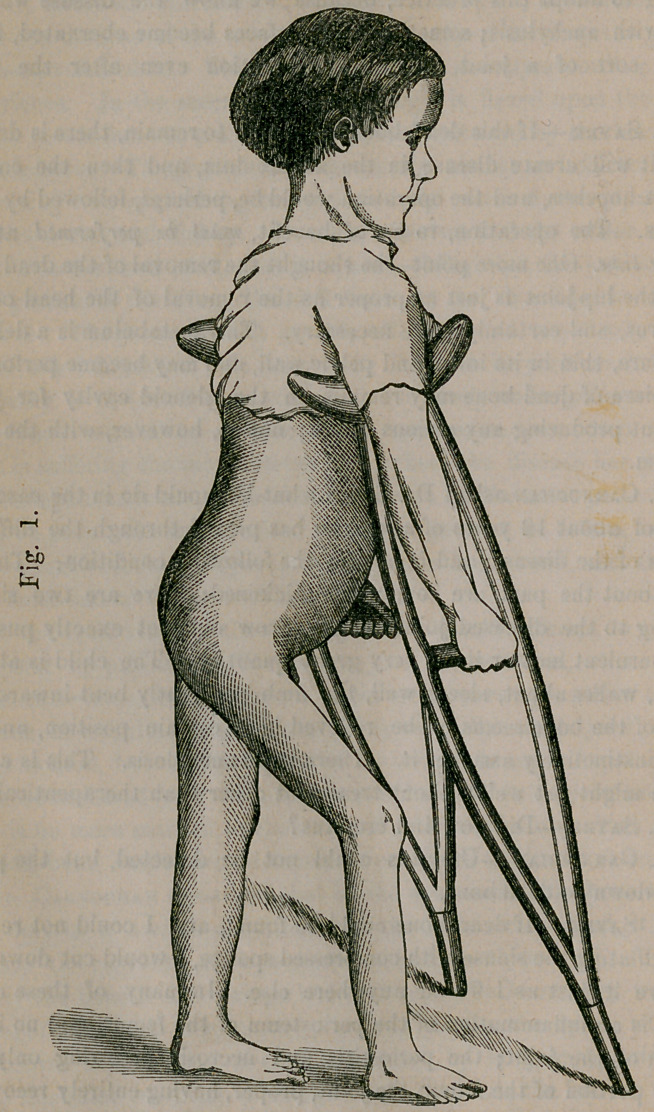


**Fig. 2. f3:**